# Predicted long-term mortality reduction associated with the second round of breast screening in East Anglia

**DOI:** 10.1054/bjoc.2000.1609

**Published:** 2001-02

**Authors:** J McCann, S Duffy, N Day, on behalf of the East Anglian breast screening programme (Director of Quality Assurance: R Warren)

**Affiliations:** Cancer Intelligence Unit, University of Cambridge Institute of Public Health, Strangeways Research Laboratory, Worts Causeway, Cambridge, CB1 8RN, UK; Department of Mathematics, Statistics and Epidemiology, Imperial Cancer Research Fund, PO Box 123, Lincoln's Inn Fields, London, WC2A 3PX, UK; Strangeways Research Laboratory, Worts Causeway, Cambridge, CB1 8RN, UK

**Keywords:** breast cancer screening, prognosis, detection mode, mortality

## Abstract

Randomized trials have demonstrated that mammographic screening can reduce breast cancer mortality. Our aim was to estimate the reduction in mortality expected from the East Anglian breast screening programme. Breast screening achieves benefit by improving cancer prognosis (reducing tumour size, nodal involvement and possibly grade) through earlier diagnosis. We compared cancer prognosis between women invited for screening and those not yet invited in East Anglia, UK, in order to predict the mortality reduction achievable by screening, independently of any reduction due to changes in treatment and underlying disease. Participants (both invited and not-yet invited) were women eligible for invitation to first and second screens and diagnosed with invasive breast cancer in 1989–96. Death rates were predicted based on the observed distribution of tumour grade, size and node status amongst 950 cancers diagnosed following first invitation, up to and including at second screen (excluding those detected at first screening), and 451 cancers presenting symptomatically in women awaiting first invitation during the staggered introduction of screening, after adjustment for lead time amongst screen detected cases. For all ages, the ratio of predicted breast cancer mortality in the invited compared with the uninvited group was 0.85 (95% CI 0.78, 0.93). It was 0.93 (0.80, 1.08) for women aged 50–54 at diagnosis and 0.81 (0.72, 0.91) for those aged 55–64. We conclude that, by 2004, the second round of screening in East Anglia should reduce mortality by around 7% in women below age 55 at diagnosis, and by around 19% in those aged 55–64. © 2001 Cancer Research Campaign http://www.bjcancer.com


*British Journal of Cancer* (2001) **84**(3), 423–428
© 2001 Cancer Research Campaign
doi: 10.1054/ bjoc.2000.1609, available online at http://www.idealibrary.com on IDEAL®

http://www.bjcancer.com

### Predicted long-term mortality reduction associated with the second round of breast screening in East Anglia

**J McCann[1], S Duffy[2] and N Day[3] on behalf of the East Anglian breast screening programme (Director of Quality Assurance: R Warren)**

[1]Cancer Intelligence Unit, University of Cambridge Institute of Public Health, Strangeways Research Laboratory, Worts Causeway, Cambridge CB1 8RN;
[2]Department of Mathematics, Statistics and Epidemiology, Imperial Cancer Research Fund, PO Box 123, Lincoln's Inn Fields, London WC2A 3PX;
[3]Strangeways Research Laboratory, Worts Causeway, Cambridge CB1 8RN, UK

**Summary** Randomized trials have demonstrated that mammographic screening can reduce breast cancer mortality. Our aim was to estimate the reduction in mortality expected from the East Anglian breast screening programme. Breast screening achieves benefit by improving cancer prognosis (reducing tumour size, nodal involvement and possibly grade) through earlier diagnosis. We compared cancer prognosis between women invited for screening and those not yet invited in East Anglia, UK, in order to predict the mortality reduction achievable by screening, independently of any reduction due to changes in treatment and underlying disease. Participants (both invited and not-yet invited) were women eligible for invitation to first and second screens and diagnosed with invasive breast cancer in 1989–96. Death rates were predicted based on the observed distribution of tumour grade, size and node status amongst 950 cancers diagnosed following first invitation, up to and including at second screen (excluding those detected at first screening), and 451 cancers presenting symptomatically in women awaiting first invitation during the staggered introduction of screening, after adjustment for lead time amongst screen detected cases. For all ages, the ratio of predicted breast cancer mortality in the invited compared with the uninvited group was 0.85 (95% CI 0.78, 0.93). It was 0.93 (0.80, 1.08) for women aged 50–54 at diagnosis and 0.81 (0.72, 0.91) for those aged 55–64. We conclude that, by 2004, the second round of screening in East Anglia should reduce mortality by around 7% in women below age 55 at diagnosis, and by around 19% in those aged 55–64. © 2001 Cancer Research Campaign http://www.bjcancer.com

**Keywords**: breast cancer screening; prognosis; detection mode; mortality

The aim of cancer screening programmes is to reduce mortality. Randomized controlled trials have shown that mammographic screening can reduce breast cancer mortality by 20–30% (Tabar et al, 1985), the benefit deriving from the reduction in tumour size, extent of axillary node involvement and possibly malignancy grade associated with earlier diagnosis (Chen et al, 1997; Tabar et al, 1999). Thus the effectiveness of screening is already established. The question of interest now, however, is the extent to which the benefit can be achieved in the population setting by a given screening programme. Specifically how does the performance of the East Anglian regional programme compare with the results obtained in the trials?

Introduced in 1989, the NHS breast screening programme (NHSBSP) aims to reduce breast cancer mortality in the invited population by at least 25% by the end of the year 2000 (Secretary of State for Health, 1992). However, assessing the impact that screening has on the target population is difficult. Simply monitoring breast cancer mortality before and after the introduction of screening is unsatisfactory because factors other than screening also influence mortality. Improvements in treatment (Beral et al, 1995; Peto et al, 2000), earlier diagnosis resulting from increased breast awareness (Stockton et al, 1997), and changes in the underlying disease due to variation in exposure to breast cancer risk factors (e.g. oral contraceptives, number of children, age at first birth etc.) (Henderson et al, 1996) influence incidence and therefore mortality. Furthermore, even given a suitable unscreened comparison group, assessing screening programme impact by monitoring deaths is unsatisfactorily slow because breast cancer survival is relatively high (Coleman et al, 1999). Mortality amongst invited women in the Swedish two-county study was not significantly lower for 6 or 7 years (Tabar et al, 1987); similar information for the UK programme will be delayed further owing to the staggered introduction of screening (McCann et al, 1998).

*Received 13 July 2000*
*Revised 11 September 2000*
*Accepted 25 October 2000*

*Correspondence to:* J McCann

When evaluating screening programmes, we must therefore address two issues: 1) how to measure only changes in mortality specifically attributable to screening and, 2) how to avoid the delay and uncertainty associated with measuring deaths.

Addressing the first issue, we require two groups of women with similar exposure to breast cancer risk factors (age, social class, number of children, etc.) but different exposure to screening invitation (i.e. one invited group, one uninvited). Treatment will be similar between groups if cancers are diagnosed within the same time period, thus any difference in outcome should be attributable to screening programme activity. Such groups were available in East Anglia when the programme started due to its staggered introduction across the region, since each district within the region set up its own screening centre individually. The first centre to start screening in the region did so early in 1989, completing the first round in 1993, and the second round in 1996. The last centre commenced two years later, in 1991, finishing the first round in 1995, and the second in 1998. It thus took 6 years to complete an entire first round in the region. Furthermore, women were invited by year of birth, in 5-year age bands, over a 3-year cycle starting with the oldest (McCann et al, 1998). Thus, although between 1989 and 1996 all but one screening unit had completed two rounds of screening, a substantial number of cancers were diagnosed in women awaiting their first invitation. In this period, one birth cohort of women eligible for at least two screening rounds therefore provided us with two comparable groups of cancers: one diagnosed in women already invited for screening and the other diagnosed in those awaiting invitation.

Addressing the second issue – one alternative to direct observation of mortality is to compare the prognosis of cancers between invited and uninvited groups. Together with survival information, the difference in prognosis may be used to estimate a difference in mortality. Results from randomized screening trials have shown that the prognostic factors tumour size, node status and malignancy grade can be used accurately to predict subsequent mortality (Tabar et al, 1995).

Using data from the East Anglian programme, we have compared the distribution of prognostic factors in two groups of cancers; one diagnosed before, and one after, first invitation for screening. We have then determined the reduction in mortality predicted by the observed difference in prognosis between these two groups. Here, we describe the shift in prognosis that has been brought about by screening and report the associated mortality reduction expected to result from the second round of breast screening in East Anglia.

### METHODS

We studied cancers in both invited and uninvited study groups, diagnosed over the period 1.1.89–31.12.96, in women born 1925–43 and eligible for invitation to the first and second rounds of the East Anglian breast screening programme. Our aim was to compare two groups of newly incident cancers. In the uninvited group cancers which arise are, by definition, newly incident since there is no screening. In the invited group we can construct a set of newly incident cancers by excluding the prevalent cases diagnosed at the initial screen, and including those diagnosed during one screening cycle i.e. from the time immediately after the first scheduled screening appointment to the time immediately following the second. This yields a set of incident tumours from a complete incidence cycle of a screening programme (interval cancers plus cancers in non-attenders plus cancers detected at the second screening): the so-called 'unbiased set' (Tabar et al, 1992). These cancers were identified as described previously (McCann et al, 1998). In this manner we have excluded cancers detected at the initial screen, which include a disproportionate number of slow growing cancers of good prognosis (so called 'length bias' (Morrison, 1992)).

Eligible women were identified on the breast screening computer system. Of those invited to first screen, around 27% exceeded the upper age limit for invitation (65 years) at the time a second screen would have been due. These were excluded from the analysis, as were women who failed to respond to the initial invitation but who attended for the first time upon reinvitation, 3 years later since, for the latter group, the scheduled 3-year repeat screen would, in fact, be an initial (prevalence) screen.

Since the introduction of screening was staggered by district and by year of birth, as described earlier, there was a substantial number of women in the region in the age group targeted for screening who were not actually invited for mammography until some years after the beginning of 1989. Cancers diagnosed in such women before their first invitation to screening formed the uninvited group.

Information on the prognostic characteristics size (maximum diameter of invasive component), grade (classified according to ICD-0 (World Health Organisation, 1990)) and node status were obtained from the Cancer Registry, breast screening units and medical records.

Predicting mortality based on prognosis requires knowledge of the baseline (underlying) survival and on the age-adjusted effects of tumour size, grade and node status on this baseline survival. We obtained this information using Cox's proportional hazards survival analysis (Cox, 1972) of a reference cancer population: the entire East Anglian study set of 1401 invasive cancers. We also repeated the analysis using a separate reference population (1528 invasive cancers diagnosed in the period 1977–88 from both arms of the Swedish Two-County trial in women aged 50–69 at randomization, of which 768 were screen-detected and 760 symptomatic).

For the reference population, we estimated a baseline survival at 88 months. A 'prognostic score' was then estimated for different combinations of categories of tumour size, grade and node status (including 'missing') using this baseline and the relevant hazard ratios (Tabar et al, 1995). This prognostic score indicated the overall probability of death for a cancer case at 88 months based on its size, grade and node status. Total deaths expected in the invited and uninvited groups were calculated by combining the number of cancer cases in each category with the prognostic score estimated for that category. Adding deaths over all categories within each group gave the total predicted deaths (Tabar et al, 1995). Relative mortality was obtained by dividing the predicted death rate (= deaths ÷ cancers) in the invited group by that predicted in the uninvited group. Confidence intervals were estimated assuming a multinomial distribution for the 112 possible prognostic classes (7 size × 4 node status × 4 grade) (Day and Duffy 1996).

Predition of mortality from the tumour size, grade and node status has been repeatedly validated and has been shown to be accurate in terms of both absolute numbers of deaths and of the relative risk of death for an invited group compared with a group not invited for screening (Organising committee and collaborators, 1996).

For screen-detected cancers, survival times are artificially increased by 'lead time' (Morrison, 1992), since their diagnosis has been advanced by screening. If earlier treatment conferred no additional benefit whatsoever over later treatment, women with screen detected cancers would die at exactly the same point in time as they would have, had they not been screened. They would still, however, show longer survival from diagnosis, the increase in survival time representing the amount of time by which screening advanced the time of diagnosis. The length of this lead time in the East Anglian programme is unknown but, in the Swedish two-county study, is estimated to be around 3 years in these age groups (Tabar et al, 1995). Given the relative detection rates in the Swedish two-county study and the East Anglian programme (Day et al, 1995) it is unlikely that the average lead time in the East Anglian programme falls outside the 24–36 months range. To adjust for it when calculating relative mortalities, baseline survival for screen detected cancers was estimated, not at 88 months, but at 124 months, corresponding to adjustment for 36 months of lead

 © 2001 Cancer Research Campaign time. Adjustment for an intermediate lead time (24 months) was also made.

### RESULTS

Of 203 194 women born between 1925–43 and invited to the first screening round, 140 387 were reinvited to second screen. A total of 451 invasive cancers occurred after 1 January 1989 and before receipt of first invitation to screening. These formed the uninvited group. 571 invasive cancers were detected at first screen and were excluded from the analysis. During the study period, in the invited group, there were 382 interval cancers presenting after first screen, 156 cancers in non-attenders at first screen, and 412 cancers detected at second screen: a total of 950 invasive breast cancers. The total study set (invited plus uninvited) therefore comprised 1401 cancers. The numbers of women invited and screened at the second screen are given by age at first invitation in Table 1, which also shows numbers of cancers diagnosed in each study group, by age at diagnosis. The mean age (standard error) at diagnosis for women in the uninvited group was 55.3 (0.16; range 50.0–63.9) and in the invited group was 58.5 (0.12, range 50.2–65.1).

The distributions of tumour size, grade and node status in each study group are shown in Table 2 and Figure 1. Tumour grade, size and node status were all more favourable in the invited group. The relative risks of death by size, grade and node status, for the East Anglian reference group, are given in Table 3: these increased with increasing tumour size, grade and node involvement. Numbers of deaths predicted in each study group, and estimated relative mortalities, are given in Table 4. After adjusting for 36 months' lead time amongst screen-detected cancers, estimated relative mortality in invited women aged 50–64 at diagnosis, compared with uninvited women, was 0.85 (95% CI 0.78, 0.93), see Table 4. For women aged 50–54, the corresponding relative mortality was 0.93 (0.80, 1.08); and for those aged 55–64, it was 0.81 (0.72, 0.91). Very similar reductions were predicted by using prognostic hazards from the Swedish reference population (data not shown).

**Table 1** Women invited and screened in the second screening round, by age *at first* invitation, and cancers arising in invited and uninvited study groups, by age at diagnosis

| Invited group | | | Whole study group | | | | | |
|---|---|---|---|---|---|---|---|---|
| | | | | Cancers in uninvited group | Cancers in invited group | | | |
| Age at first invitation | Women invited to second screen | Women screened at second screen | Age at diagnosis | | Intervals | Non-attenders | Second screen detected | Invited group total |
| <50 | 3085 | 2331 | <50 | 0 | 0 | 0 | 0 | 0 |
| 50–54 | 55 444 | 41 685 | 50–54 | 225 | 114 | 38 | 54 | 206 |
| 55–59 | 56 439 | 41 002 | 55–59 | 184 | 150 | 64 | 164 | 378 |
| 60–64 | 25 419 | 16 003 | 60–64 | 42 | 116 | 54 | 94 | 364 |
| 65–69 | 0 | 0 | 65–69 | 0 | 2[a] | 0 | 0 | 2 |
| <50–64 | 140 387 | 101 021 | 50–64 | 451 | 382 | 156 | 412 | 950 |

[a]These cancers occurred in women aged 62.1–62.3 at first screening.

**Table 2** Numbers of cancers and percent of cases with known information in invited and uninvited study groups according to tumour size, grade and node status

| | | Study groups | | | | | | | | | | | |
|---|---|---|---|---|---|---|---|---|---|---|---|---|---|
| | | Invited | | | | | | Uninvited | | | | | |
| Prognostic characteristic | Category | <55 | | 55+ | | all ages | | <55 | | 55+ | | all ages | |
| | | *n* | % | *n* | % | *n* | % | *n* | % | *n* | % | *n* | % |
| Size (mm) | 1–9 | 25 | 13 | 110 | 16 | 135 | 15 | 8 | 4 | 8 | 4 | 16 | 4 |
| | 10–14 | 32 | 17 | 146 | 21 | 178 | 20 | 24 | 13 | 24 | 13 | 48 | 13 |
| | 15–19 | 37 | 20 | 163 | 23 | 200 | 23 | 41 | 22 | 32 | 17 | 73 | 19 |
| | 20–29 | 51 | 27 | 154 | 22 | 205 | 23 | 66 | 35 | 69 | 36 | 135 | 36 |
| | 30–49 | 28 | 15 | 93 | 13 | 121 | 14 | 34 | 18 | 38 | 20 | 72 | 19 |
| | 50+ | 16 | 8 | 28 | 4 | 44 | 5 | 17 | 9 | 19 | 10 | 36 | 9 |
| | Missing | 17 | – | 50 | – | 67 | – | 35 | – | 36 | – | 71 | – |
| | Total | 206 | | 744 | | 950 | | 225 | | 226 | | 451 | |
| Node status | Negative | 100 | 60 | 326 | 59 | 426 | 60 | 70 | 51 | 58 | 48 | 128 | 50 |
| | Positive | 60 | 36 | 197 | 36 | 257 | 36 | 60 | 44 | 57 | 48 | 117 | 46 |
| | Metastases | 6 | 4 | 25 | 5 | 31 | 4 | 7 | 5 | 5 | 4 | 12 | 5 |
| | Missing | 40 | – | 196 | – | 236 | – | 88 | – | 106 | – | 194 | – |
| | Total | 206 | | 744 | | 950 | | 225 | | 226 | | 451 | |
| Grade | 1 | 29 | 19 | 157 | 28 | 186 | 26 | 20 | 20 | 10 | 10 | 30 | 15 |
| | 2 | 78 | 50 | 272 | 49 | 350 | 49 | 49 | 49 | 51 | 50 | 100 | 50 |
| | 3 | 49 | 31 | 125 | 23 | 174 | 25 | 31 | 31 | 40 | 40 | 71 | 35 |
| | Missing | 50 | – | 190 | – | 240 | – | 125 | – | 125 | – | 250 | – |
| | Total | 206 | | 744 | | 950 | | 225 | | 226 | | 451 | |

*© 2001 Cancer Research Campaign*

**Table 3** Estimates of relative hazards (with 95% confidence intervals) based on proportional hazards regression, with each factor adjusted for the others and for age group, for all invasive cancers in the East Anglian reference group

| Prognostic characteristic | Adjusted relative hazard (95% CI) |
|---|---|
| Size (mm) | |
| 1–9 | 0.37 (0.17, 0.80) |
| 10–14 | 0.52 (0.32, 0.85) |
| 15–19 | 0.87 (0.61, 1.25) |
| 20–29 | 1.00[a] |
| 30–49 | 1.34 (1.97, 1.85) |
| 50+ | 2.28 (1.56, 3.33) |
| missing | 1.27 (0.88, 1.83) |
| Node status | |
| negative | 1.00[a] |
| positive | 3.88 (2.90, 5.38) |
| metastases | 11.60 (7.30, 18.45) |
| missing | 2.04 (1.44, 2.89) |
| Grade | |
| 1 | 0.29 (0.15, 0.58) |
| 2 | 1.00[a] |
| 3 | 1.50 (1.09, 2.06) |
| missing | 1.22 (0.27, 1.63) |

[a]Reference category. The baseline survival predicted by the regression for the reference group was 0.88 at 88 months (no adjustment for lead time) and 0.83 at 124 months (adjusting for 36 months lead time), for Grade 2, node negative, size 20–29 mm tumours.

Greater mortality reductions were predicted if adjustment for a shorter lead time was made (adjustment for 24 months' lead time gave a relative mortality (95% CI) of 0.81 (0.74, 0.89) for women aged 50–64; 0.91 (0.78, 1.06) for those aged <55, and 0.76 (0.68, 0.84) for those aged 55–64).

### DISCUSSION

As a consequence of the more favourable tumour size, grade and node status of cancers in the invited study group, we predict that mortality amongst breast cancers diagnosed in women aged 50–64 following invitation for screening will be at least 15% (95% CI 7, 22%) lower than for cancers diagnosed in the same population before invitation to screening.

For women aged 55 or older at diagnosis, the predicted reduction is greater (19%), whereas for women aged under 55, it is small (7%) and not significant, as noted previously in other screened populations and attributed to the reduced sensitivity of screening and faster rate of tumour progression in these younger women (Tabar et al, 1995; Duffy et al, 1996). These predictions may underestimate the true impact of screening, since the 3 years' lead time allowed to adjust for the earlier diagnosis of screen detected cancers in the invited group may be an overestimate. Allowing just 2 years' lead time for the screen detected cases would predict a 19% mortality reduction in the entire age group, and a 24% reduction in women aged over 55 at diagnosis.

A recent paper (Blanks et al, 2000) has estimated using observed mortality rates that in 1998, in women aged 55–69, the reduction in breast cancer mortality due to screening was 6.4%. This reduction derives from screening at the initial round. Our results refer to screening after the initial round and predict the reduction in breast cancer mortality in the future. They suggest an improvement in the performance of the screening programme after the initial round, already noted nationally in increased cancer detection rates (Young et al, 1997).

Women in the invited group were, on average, 3 years older at diagnosis than those in the uninvited group. This difference arose because older women were invited for screening first, giving less time in which cancers could present before invitation. This difference in age should not affect results, however, because we adjusted for age group at diagnosis when estimating hazards associated with prognostic characteristics, and have also presented age-stratified results. The age-adjusted relative mortality is identical with the unadjusted (i.e. 0.85) with a slightly wider 95% confidence interval (0.77, 0.94).

Our results indicate that the second round of the East Anglian breast screening programme is likely to deliver a 15% reduction in breast cancer mortality, falling short of the 25% reduction expected from the randomized trials. In the age group 55–64, however, a reduction more in line with the trial results is predicted. It should be understood, however, that these results pertain to the first few years of the programme. It may be that changes in practice at screening (e.g. higher film density) and at assessment (e.g. greater experience of percutaneous biopsy) may have improved the sensitivity of screening, thus more recent results might demonstrate a greater benefit. The results here, however, are the most reliable available for the East Anglian programme and they suggest room for improvement, notably in women aged under 55. Table 2 suggests that sensitivity to small tumours needs to be improved in these younger women. Some improvements have

**Table 4** Total deaths predicted after 88 months based on tumour size, histologic grade and lymph node status, using relative hazards from Table 3, with adjustment for 3 years of lead time

| Age | Study group | Breast cancer cases | Total deaths predicted | Predicted deaths per 100 cases | Relative mortality (95% CI) |
|---|---|---|---|---|---|
| <55 | Uninvited | 225 | 65.87 | 29.3 | 0.93 |
| | Invited | 206 | 56.34 | 27.3 | (0.80, 1.08) |
| 55+ | Uninvited | 226 | 71.13 | 31.5 | 0.81 |
| | Invited | 744 | 188.62 | 25.4 | (0.72, 0.91) |
| 50–64 | Uninvited | 451 | 137.01 | 30.4 | 0.85 |
| | Invited | 950 | 244.95 | 25.8 | (0.78, 0.93) |

© 2001 Cancer Research Campaign

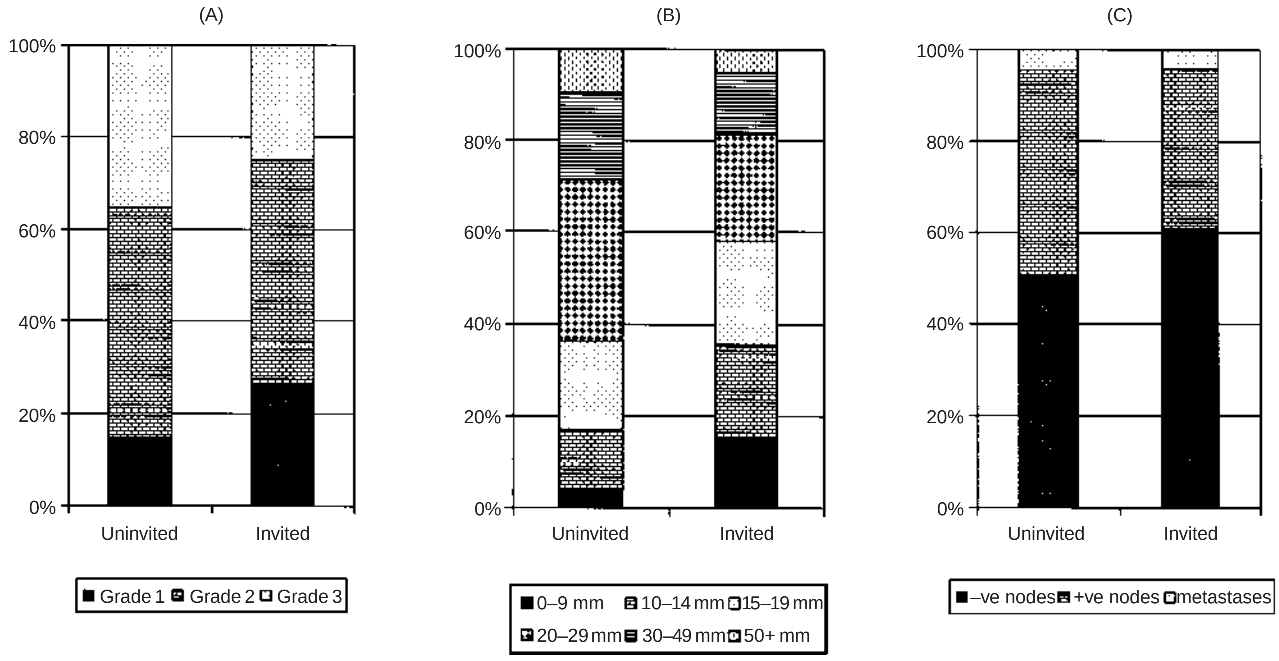

**Figure 1** Distribution of tumour grade (**A**), size (**B**) and node status (**C**), as proportion of total cases with known information, in invited and uninvited study groups already been made with the change towards using higher mammographic film density (Young et al, 1997).

There are advantages in predicting – rather than directly observing – changes in mortality. Firstly, results are available soon after completion of the second screening round; several years before sufficient mortality data have accrued. Secondly, the evaluation considers essentially a single population of women, before and after invitation to screening: cancers in the invited and uninvited study groups are diagnosed over essentially the same time period, and results are based on pathological measures of disease extent at diagnosis. They are therefore unaffected by temporal changes in treatment or in changing incidence or stage at presentation caused either by variation in the underlying risk of disease or by participation bias due to socioeconomic or other differences. A final strength of our approach is the increased power obtained by using predicted mortality, hence the narrow confidence intervals for the relative mortalities in Table 4. If the relative risk of 0.85 for the age group 50–64, with 36 months lead time, had been based on 137 and 245 *observed* deaths, the confidence interval would be 0.72, 1.01. Using *predicted* deaths, the confidence interval is roughly half the width. We can thus estimate more precisely the magnitude of the impact of screening on mortality (Day and Duffy, 1996).

The main drawback to using predicted mortality for assessing screening programme performance is the reliance on the underlying model. However the approach we have used here has been shown to predict mortality in the Swedish two-county study with considerable accuracy (Tabar et al, 1995). Furthermore, although the predicted effect on mortality is determined by the effects on survival of tumour size, grade and node status estimated using East Anglia data, we obtained very similar estimates of predicted mortality reduction using a completely separate source (the Swedish two-county study) for survival information based on prognostic factors. This concordance supports the robustness of the method.

Recently population-based breast screening programmes have come under attack with highly publicized reports of lack of effectiveness (Sjönell and Ståhle, 1999; Gøtzsche and Olsen, 2000). In welcome contrast our results indicate that, despite rates for interval cancers that were initially higher than expected (Day et al, 1995; Woodman et al, 1995), the second round of screening in East Anglia should deliver a substantial mortality reduction through its effects on tumour size, grade and node status. Given similar distributions of prognostic characteristics amongst cancers in screened and non-screened women, we would expect other regional programmes to achieve similar impact, although clearly there may be some variation between regional programmes due to differences in screening performance and effectiveness of treatment. However we estimate that, independently of any reduction expected due to changes in treatment and underlying disease, screening throughout the East Anglian region should reduce breast cancer deaths by around a further 19% in women aged 55–64 at diagnosis by the year 2004.

### ACKNOWLEDGEMENTS

JM was funded by a grant from the National Health Service executive of the Eastern Region office and by the East Anglian Breast Screening quality assurance programme.

The authors wish to thank directors and staff at the region’s 7 screening units, 3 health authorities and the East Anglian Cancer Registry for their co-operation and support in providing data. They are also grateful to Peter Treasure, Diane Stockton and Sara Godward for valuable discussions of the method and help with software. They thank Neil Walker for writing the computer matching program, and Ruth Warren and Tom Davies for their helpful comments during preparation of the manuscript.

*© 2001 Cancer Research Campaign* 

**REFERENCES**

Beral V, Herman C, Reeves G and Peto R (1995) Sudden fall in breast cancer death rates in England and Wales. *Lancet* **345**: 1642–1643

Blanks RG, Moss SM, McGahan C, Quinn M and Babb PJ (2000) Effect of NHS breast screening programme on mortality from breast cancer in England and Wales 1990–98: Comparison of observed with predicted mortality. *Br Med J* **321**: 665–669

Chen HH, Duffy S, Tabar L and Day NE (1997) Markov chain models for the progression of breast cancer, part 1: tumour attributes and the preclinical screen-detectable phase. *J Epidemiol Biostat* **2**: 9–23

Coleman MP, Babbs P, Damiecki P, Grosclaude P, Honjo S, Jones J, Knerer G, Pitard A, Quinn M, Sloggett A and De Stavola B (1999) *Cancer survival trends in England and Wales 1971–1995: deprivation and NHS region.* Office for National Statistics: London

Cox DR (1972) Regression model and life tables. *J R Stat Soc B* **34**: 187–220

Day NE and Duffy S (1996) Trial design based on surrogate endpoints: application to a trial of different breast screening frequencies. *J R Stat Soc A* **159**: 49–60

Day NE, McCann J, Camilleri-Ferrante C, Britton P, Hurst G, Cush S and Duffy S (1995) Monitoring interval cancers in breast screening programmes: the East Anglian experience. *J Med Screening* **2**: 180–185

Duffy S, Chen HH, Tabar L, Fagerberg G and Paci E (1996) Sojourn time, sensitivity and positive predictive value of mammography screening for breast cancer in women aged 40–49. *Int J Epidemiol* **25**: 1139–1145

Gøtzsche PC and Olsen O (2000) Is screening for breast cancer with mammography justifiable? *Lancet* **355**: 129–134

Henderson BE, Pike MC, Bernstein L and Ross RK (1996) Breast cancer. In: *Cancer epidemiology and prevention.* Schottenfeld D and Fraumeni Jr JF (eds), Oxford University Press: New York, Oxford

McCann J, Stockton D and Day NE (1998) Breast cancer in East Anglia: the impact of the breast screening programme on stage at diagnosis. *J Med Screening* **5**: 42–48

Morrison AS (1992) Early detection: sensitivity and lead time. In: *Screening in Chronic Disease*, pp 43–73. Oxford University Press: New York, Oxford

Organising committee and collaborators FM (1996) Breast cancer screening with mammography in women aged 40–49 years. *Int J Cancer* **68**: 693–699

Peto R, Boreham J, Clarke M, Davies C and Beral V (2000) UK and USA breast cancer deaths down 25% in year 2000 at ages 20–69 years. *Lancet* **355**: 1822–1830

Secretary of State for Health (1992) *The Health of the Nation. A strategy for health in England*. London, HMSO.

Sjönell G and Ståhle L (1999) Mammography screening does not significantly reduce breast cancer mortality in Swedish daily practice. *Lakartidningen* **96**: 904–913

Stockton D, Davies T, Day NE and McCann J (1997) Retrospective study of reasons for improved survival in patients with breast cancer in East Anglia: earlier diagnosis or better treatment? *BMJ* **314**: 472–475

Tabar L, Gad A, Holmberg LH, Ljungquist U, Eklund G, Fagerberg CJG, Baldetorp L, Gröntoft O, Lundström B, Månson JC and Day NE (1985) Reduction in breast cancer mortality by mass screening with mammography: first results of a randomised trial in two Swedish counties. *Lancet* **1**: 829–832

Tabar L, Fagerberg CJG, Day NE and Holmberg L (1987) What is the optimum interval between mammographic screening examinations? An analysis based on the latest results of the Swedish two county breast cancer screening trial. *Br J Cancer* **55**: 547–551

Tabar L, Fagerberg CJG, Duffy S, Day NE, Gad A and Grontoft O (1992) Update of the Swedish two-county program of mammographic screening for breast cancer. *Radiol Clin North Am* **30**: 187–209

Tabar L, Fagerberg G, Chen HH, Duffy S, Smart C, Gad A and Smith RA (1995) Efficacy of breast cancer screening by age. *Cancer* **75**: 2507–2517

Tabar L, Duffy S, Chen HH, Vitak B and Prevost T (1999) The natural history of breast carcinoma. What have we learned from screening? *Cancer* **86**: 449–462

Woodman CBJ, Threlfall AG, Boggis CRM et al (1995) Is the three year breast screening interval too long? Occurrence of interval cancers in the NHS breast screening programme's north western region. *BMJ* **310**: 224–226

World Health Organisation (1990) *International Statistical Classification of Diseases and Related Health Problems*. World Health Organisation: Geneva

Young KC, Wallis MG, Blanks RG and Moss SM (1997) Influence of number of views and mammographic film density on the detection of invasive cancers: results from the NHS breast screening programme. *British Journal of Radiology* **70**: 487–488

*© 2001 Cancer Research Campaign*
